# The Influence of Physical Activity and Positive Affect on the Relationship between Pain Severity and Cognitive Performance in Women with Fibromyalgia

**DOI:** 10.3390/jcm13154419

**Published:** 2024-07-28

**Authors:** Patricia Catalá, Lorena Gutiérrez, Carmen Écija, Cecilia Peñacoba

**Affiliations:** Department of Psychology, Rey Juan Carlos University, Avda. de Atenas s/n, 28922 Alcorcón, Madrid, Spain; patricia.catala@urjc.es (P.C.); lorena.gutierrezh@urjc.es (L.G.); carmen.ecija@urjc.es (C.É.)

**Keywords:** fibromyalgia, chronic pain, cognitive performance, moderate physical activity, positive affect

## Abstract

Fibromyalgia, characterized by chronic pain and cognitive impairments, significantly impacts patients’ quality of life. Physical exercise has been shown to improve cognitive functions and reduce pain severity. Additionally, positive affect enhances cognitive flexibility and facilitates better adaptation to chronic pain, suggesting that combining physical activity and positive affect could mitigate cognitive deficits in fibromyalgia patients. **Objectives:** The objective of this work is to explore the relationship between pain severity and cognitive performance through time spent doing moderate physical activity while taking into account the role of positive affect in fibromyalgia. **Methods:** This prospective study was structured into two phases of evaluation. First, pain severity, positive affect, and time spent performing moderate physical activity were evaluated, and one week later cognitive performance (assessed through the Stroop test) was also evaluated. The final sample consisted of 231 women with fibromyalgia. Moderated mediation analyses were performed using PROCESS. **Results:** The moderated mediation model showed that the effect of moderate physical activity on the relationship between pain severity and cognitive performance was significant for low levels of positive affect but not for moderate or high levels. That is, the indirect effect of pain intensity on cognitive performance through time spent doing moderate physical activity only has an effect when patients with fibromyalgia present low levels of positive affect. However, there was no significant indirect effect in the simple mediation model. **Conclusions:** The findings of this study underscore the importance of considering the level of positive affect when examining the impact of moderate physical activity on cognitive performance in women with fibromyalgia.

## 1. Introduction

Fibromyalgia is a chronic disorder characterized by widespread pain, fatigue, and a variety of physical and cognitive symptoms [1]. Among these symptoms, cognitive deficits, commonly referred to as “fibrofog”, include difficulties related to attention, memory, and executive functions [2]. These cognitive impairments significantly impact patients’ quality of life, making daily and professional activities difficult [3,4].

Furthermore, the experience of chronic pain, considered a main symptom of fibromyalgia, is strongly linked to impairments in cognitive performance [5]. Previous research has shown that pain can interfere with attentional and working memory processes, resulting in lower performance in cognitive tasks [6]. This phenomenon can be explained by the “limited workspace” theory, where pain processing consumes cognitive resources that would otherwise be available for other tasks [7].

In this regard, physical exercise has been identified as an effective intervention to improve cognitive function in diverse populations, including those with chronic pain disorders [8]. In patients with fibromyalgia, regular exercise can improve attention and memory, possibly by increasing cerebral circulation and reducing inflammation and oxidative stress [9,10,11]. A longitudinal study revealed that a 12-month exercise program resulted in significant improvements in Stroop test scores, a common measure of executive function [12].

In addition to physical exercise, positive affect, defined as the degree of positive emotion a person experiences, also plays a crucial role in cognitive function [13]. It has been shown that positive affective states can broaden the repertoire of thoughts and actions, thereby improving cognitive flexibility and problem-solving abilities [14]. In the context of chronic pain, greater positive affect has been associated with better adaptation to pain and less impact of pain in daily life [15,16].

Physical activity not only improves cognitive function directly but can also increase positive affect, creating a beneficial cycle [17]. For example, regular participation in physical activities can elevate levels of neurotransmitters such as dopamine and serotonin, which are related to emotional well-being [18,19]. Furthermore, exercise can provide a sense of accomplishment and control, contributing to greater positive affect and subsequently better cognitive function [20].

Several studies have shown that positive affect can be both a consequence and a facilitator of physical exercise. For example, Berger and Motl [21] found that people who participate in moderate physical activity report an increase in positive affect after exercise. Furthermore, longitudinal studies have shown that individuals with higher levels of positive affect are more likely to maintain an exercise routine in the long term [22].

The interaction between moderate physical activity and positive affect, and the effect this has on cognitive performance, may be particularly relevant for the chronic pain population, and especially in patients with fibromyalgia. Therefore, the present study aims primarily at exploring the relationship between pain severity and cognitive performance through time spent undertaking in moderate physical activity, taking into account the role of positive affect in fibromyalgia. Based on the existing literature, it is hypothesized that both moderate physical exercise and greater positive affect may attenuate the negative effects of pain on cognition, thus improving the quality of life of patients. Specifically, the following hypotheses are proposed: (1) moderate physical activity will mediate the relationship between pain severity and cognitive performance in women with fibromyalgia; (2) positive affect will moderate the relationship between pain severity and moderate physical activity, such that high levels of positive affect will strengthen the relationship between lower pain severity and greater participation in moderate physical activity; and (3) positive affect will also moderate the relationship between moderate physical activity and cognitive performance, such that high levels of positive affect will enhance the beneficial effects of moderate physical activity on cognitive performance.

## 2. Materials and Methods

### 2.1. Participant

According to global statistical data, it is estimated that 95% of patients diagnosed with FM are women, while diagnoses in men are only around 5% [23]. Therefore, a convenient sample of women diagnosed with fibromyalgia was chosen by contacting several patient associations from different Spanish regions during 2019. Following the sampling criteria for the model analysis [24], a baseline of 200 participants was set as the minimum sample size. The initial sample of the study consisted of 244 women (first evaluation moment); however, the final sample consisted of 231 women (second evaluation moment), resulting in a sample loss of 5.33%. All participants were required to meet the fibromyalgia diagnostic criteria established by the American College of Rheumatology (ACR 2010) [23,25]. Additional criteria for inclusion in the study were as follows: being female, over the age of 18, providing written informed consent, and having a medical prescription for walking with no physical limitations that would hinder physical activity.

### 2.2. Design and Procedure

This prospective study was structured in two assessment phases. The first phase consisted of the assessment of demographic and clinical characteristics, pain severity, positive affect, physical activity anxiety, and depression; in the second and final phase, a psychologist from the research team administered the Stroop test to assess the cognitive performance of the participants. The study design complied with the ethical standards for research involving human subjects, in accordance with the guidelines outlined by the Strengthening the Reporting of Observational Studies in Epidemiology (STROBE) Declaration and the Declaration of Helsinki. The research protocol received approval from the Ethics Committee of the Rey Juan Carlos University on 9 June 2016 (Reference PI17/00858).

To gather the sample, the research team contacted fibromyalgia associations in Spain. Interested associations facilitated contact with their members who met the inclusion criteria. Visits by the research team to the associations were arranged to carry out the assessments, minimizing patient travel and maximizing their participation.

At the start of each assessment phase, participants were briefed on the study’s objectives, procedures, and methods, and their written informed consent was obtained. All patients agreed to participate voluntarily and were informed that they could withdraw their consent at any time. A questionnaire booklet was provided which took approximately 30 min to complete for the first assessment and 10 min for the second assessment. Two psychologists supervised the completion of the questionnaires to ensure data integrity and to clarify any doubts.

### 2.3. Measures

#### 2.3.1. Severity Pain

To gauge the severity of pain, we averaged the scores from four pain severity items found in the Brief Pain Inventory [26]: the highest, lowest, and average pain intensity over the past 7 days and the pain intensity at the current moment. An 11-point numerical scale was used for each assessment, ranging from 0 (no pain) to 10 (the most severe pain imaginable). This method of measuring pain intensity is widely recognized in the field of pain research [27]. In this study, the scale showed high internal consistency with a Cronbach’s alpha of 0.86.

#### 2.3.2. Positive Affect

Positive affect was measured using the Positive Affect subscale of the Positive and Negative Affect Schedule (PANAS) [28]. This subscale comprises 10 items that that assess feelings of pleasurable engagement, such as joy, happiness, and enthusiasm (with a possible score range from 0 to 40). The Spanish version of this subscale has shown good reliability (α = 0.87) [29,30]. Furthermore, a recent study on chronic pain also found high reliability (α = 0.92) for positive affect [31]. In our study, the subscale showed excellent internal consistency (0.91).

#### 2.3.3. Moderate Physical Activity

To assess the physical activity levels of the study subjects at the first assessment time, the Spanish version of the International Physical Activity Questionnaire (IPAQ) was used [32,33]. This instrument allows for the quantification of physical activity at different intensities performed during the previous 7 days. In line with the IPAQ’s short and long form data processing and analysis guidelines, a uniform procedure was adhered to in order to determine the level of physical activity for each individual [34]. Physical activity levels were classified as follows: high levels (achieved by performing ≥3 days of vigorous activity, reaching ≥1500 metabolic equivalent of task minutes per week, or completing ≥7 days of any combination of walking, moderate-intensity, or vigorous-intensity activities, totaling ≥3000 metabolic equivalent of task minutes per week), moderate levels (achieved by those who performed ≥3 days of vigorous-intensity activities, walked ≥30 min per day, or performed ≥5 days of moderate-intensity activities, totaling ≥600 metabolic equivalent of task minutes per week. This level was the main focus of the study, considering the importance of maintaining regular moderate-intensity physical activity for general health), and low levels (this corresponded to participants who did not meet the criteria established for high and moderate levels). In this study, the measure of moderate levels of activity was used.

The version of the IPAQ used was previously validated within the Spanish population and showed remarkable reliability in various metrics [33]. Internal consistency, measured by Cronbach’s alpha, yielded values of 0.79 for moderate activity, indicating good reliability in regard to the questionnaire.

#### 2.3.4. Cognitive Performance

To assess cognitive performance, the Stroop Test, a standard measure of selective attention ability and cognitive inhibition, is used [35,36]. The version used consists of three parts: color word reading (participants must read aloud a list of words that are names of colors printed in black), color blob naming (participants must name the colors of a series of colored blobs), and the Stroop task (participants must name the color of the ink in which color words that do not match the name of the word are printed, such as the word “red” printed in blue ink). Both response time and errors made are recorded. For this study, the inference measure was used. This measure provides a more complete and accurate assessment of patients’ ability to inhibit automatic responses and manage cognitive interference. In addition, it helps control for potential biases that may arise from variability in basic color-reading or naming skills between individuals, focusing on the ability to manage interference. To calculate the Stroop interference measure, response times from the following three tasks are used: Color-Name Reading time (the time it takes the participant to read a list of color names printed in black), Blob-Color Naming time (the time it takes the participant to name the colors in a series of different colored “X”s), and Incongruent Stroop time (the time it takes the participant to name the color of the ink in which the color words are printed, ignoring the meaning of the words). The interference measure is calculated as the difference between the time from the Incongruent Stroop task and a weighted average of the other two tasks: Interferencia de Stroop = Tiempo SI − [(Tiempo LNC + Tiempo DCM)/2].

#### 2.3.5. Anxiety and Depression

In the study, the Spanish-adapted version of the Hospital Anxiety and Depression Scale was used [37]. This tool consists of 14 items divided into two categories: 7 for assessing anxiety and 7 for depression. Participants respond to each item on a four-point Likert scale ranging from 0 (equivalent to “no, not at all”) to 3 (meaning “yes, definitely”). A higher score indicates higher levels of anxiety and depression, with 21 being the maximum possible. In this study, the estimates of internal consistency were satisfactory for both domains, with 0.82 for anxiety and 0.85 for depression.

#### 2.3.6. Demographic and Clinical Characteristics

The research team used an ad hoc questionnaire. The following were assessed: age (in years), place of residence, relationship status (either married or in a committed relationship, single, or divorced/widowed), level of education (elementary, secondary, or higher), and occupational status (either homemakers or employed outside the home). In addition, weight, height, and the number of doses of various specific types of medication (about muscle relaxants, antidepressants, anxiolytics, and sleeping pills) that the women took in a week were recorded. That is, if a patient mentioned that she took 4 tablets of antidepressants per week, that means that she took 4 doses of a specific type of antidepressant in a week.

### 2.4. Statistical Analysis

**Figure 1:**
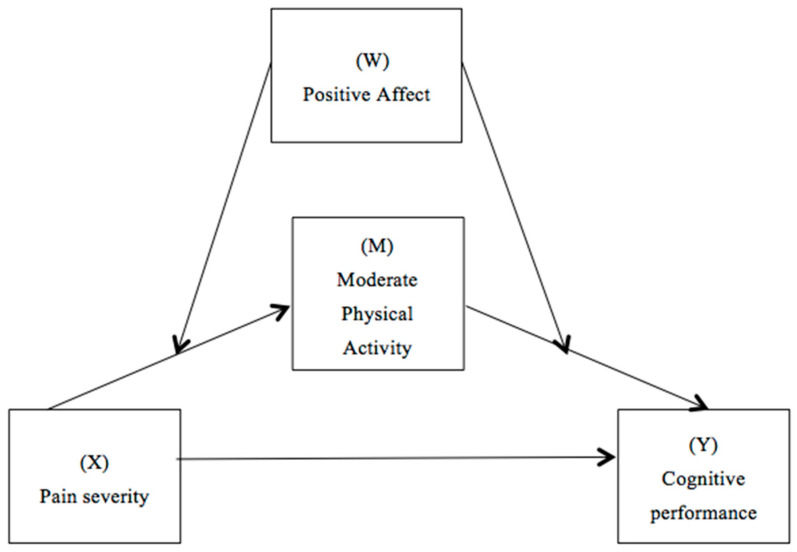
Path diagram illustrating the moderate mediation model.

For the data analysis, IBM SPSS Statistics 22.0 software [38] and PROCESS macro v3.3 for SPSS [39] were used. Descriptive analyses were conducted to determine the mean, standard deviation, and range of the sample, aiming to examine the sample’s characteristics and the distribution of the variables under study. The relationships between the main variables were analyzed using Pearson correlation coefficients. Subsequently, a moderated mediation analysis (model 58), as suggested by Hayes [39], was performed, which combines simple mediation with moderation to investigate whether the effect of the mediator variable varies across the categories of the moderator variable. This analysis explored whether the positive affect (W) alters the indirect effect of pain severity (X) on cognitive performance (Y) through moderate physical activity (M), considering as covariates the numbers of drugs taken weekly (e.g., muscle relaxants, antidepressants, analgesics, and sleeping pills), weight, height, anxiety, and depression (see Figure 1). Before incorporating these variables into the model, it was verified that they were correlated with each other. Statistical significance was defined with a two-tailed *p*-value of less than 0.01. The bootstrapping method with 5000 replications was used to establish 95% confidence intervals to assess statistical significance.

## 3. Results

### 3.1. Sample Characteristics

The average age of the study group was 56.99 years, with a variability represented by a standard deviation of 10.35 years. Of the participants, 136 (55.73%) resided in rural areas and 109 (44.27%) resided in urban areas. A minority percentage of 15% of the participants had attained a higher education level. The rest reported having completed secondary education (61%) or primary education (24%). A significant proportion, 53%, was married or in a stable relationship, while 11% remained single and 36% had been divorced or widowed. The predominant occupation among the participants was that of housewife, with 76% of the total. The mean height was 172 cm (SD = 13.3) and the mean weight was 72.13 kg (SD = 16.31). Regarding the use of medications, the average weekly use of muscle relaxants was 2.44 (SD = 3.17), of antidepressants was 4.63 (SD = 3.31), of analgesics was 5.54 (SD = 2.53), and of sleeping pills was 4.30 (SD = 3.32).

### 3.2. Correlations

Table 1 shows the relationship between several factors such as pain severity, positive affect, moderate physical activity, cognitive performance, the number of different drugs taken per week (e.g., muscle relaxants, antidepressants, analgesics, and sleeping pills), as well as weight, height, anxiety, and depression. Pain severity was inversely related to moderate physical activity (*p* = 0.018) and the cognitive performance test score (*p* = 0.34). Furthermore, moderate physical activity was directly related to positive affect (*p* = 0.004) and inversely related to the cognitive performance test score (*p* = 0.002). The cognitive performance test was inversely related to both the number of muscle relaxants (*p* = 0.028) and analgesics taken per week by the participants (*p* = 0.026). Regarding the drugs included, all were significantly related to each other (*p* < 0.05). Anxiety was significantly related to the number of antidepressants (*p* = 0.016) and sleeping pills (*p* = 0.035) taken by participants per week. Finally, depression was inversely related to positive affect (*p* = 0.001) and directly related to anxiety (*p* = 0.001) and the number of antidepressants (*p* = 0.002) and sleeping pills (*p* = 0.007) taken by participants per week.

### 3.3. Moderated-Mediation Model

**Figure 2:**
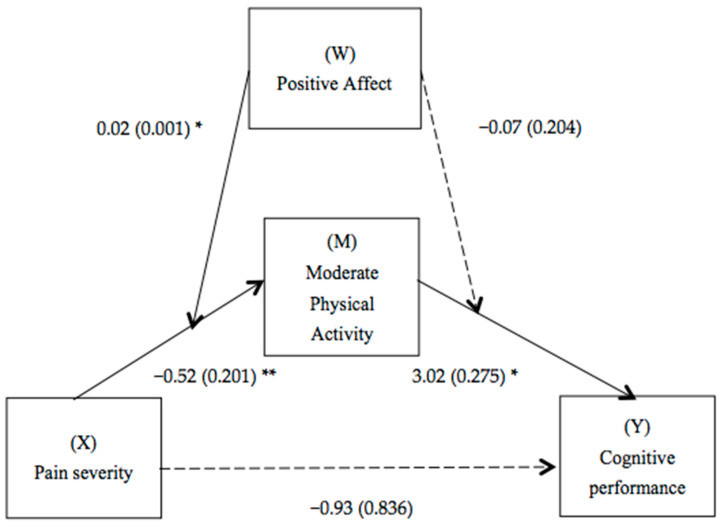
Path diagram illustrating the moderate mediation model. Notes: Values are nonstandardized regression coefficients (SE in parentheses) and associated *p* values (* *p* < 0.05, ** *p* < 0.01). Association in brackets = direct effect (controlling for indirect effects). Significant paths are indicated by solid lines; non-significant paths are indicated by dashed lines.

Table 2 shows the moderated mediation analysis considering positive affect as a moderator when moderate physical activity was used as a mediator in the relationship between pain severity and cognitive performance. The interaction between pain severity and positive affect was significant (*p* = 0.016) but the interaction between moderate physical activity and positive affect was not significant (*p* = 0.717). In addition, a direct effect of pain severity on moderate physical activity (*p* = 0.010) and of moderate physical activity on cognitive performance (*p* = 0.030) was observed. However, a direct effect of pain severity on cognitive performance was not observed (*p* = 0.268). In this study, only those covariates that showed a significant relationship with the target variables were included as covariates (e.g., antidepressants, analgesics and depression), in accordance with the recommendations for mediation and moderation analysis proposed by Hayes and by Baron and Kenny [39,40]. This selection ensures that the covariates included really contribute to the explanation of the model, avoiding the inclusion of irrelevant variables that could introduce noise in the analyses. Thus, it is observed that the mediation of moderate physical activity in the relationship between pain severity and cognitive performance was moderated by positive affect. Specifically, positive affect is found to act as a moderator in the relationship between pain severity and moderate physical activity when the latter is a mediator between pain severity and cognitive performance (see Figure 2).

The developed model is able to explain 47% of the variance in cognitive performance. To better understand the indirect effects of pain severity on cognitive performance, the bootstrap method was applied. This analysis focused on evaluating the effects at three different levels (one standard deviation below, one standard deviation above the mean, and one standard deviation at the mean) of positive affect on pain, using 95% confidence intervals to examine the indirect effects. The results, detailed in Table 3, reveal that the mediating influence of moderate physical activity varies depending on the degree of positive affect. Specifically, this mediating effect is accentuated and significant when the level of positive affect is one standard deviation below the mean. This suggests that fibromyalgia patients with high levels of pain are more likely to show poor cognitive performance despite engaging in moderate physical activity when they have low levels of positive affect.

## 4. Discussion

The results of this study provide a more nuanced understanding of the complex relationships between pain severity, moderate physical activity, positive affect, and cognitive performance in women with fibromyalgia. First, the findings indicate that pain severity has a significant direct effect on moderate physical activity. This result suggests that the greater the pain severity, the more likely patients are to reduce their level of moderate physical activity. This negative relationship is consistent with previous studies that have documented how chronic pain limits individuals’ ability to engage in physical activity [5,41,42]. Importantly, given the negative impact of pain on physical activity, pain management strategies that enable patients to maintain an adequate level of physical activity should be considered.

A significant direct effect of moderate physical activity on cognitive performance was also observed. This finding supports previous evidence suggesting that physical activity may improve cognitive function, possibly by increasing cerebral circulation and reducing inflammation and oxidative stress [8,9,43]. Therefore, on a clinical level, encouraging moderate physical activity in patients with fibromyalgia could be an effective strategy to mitigate the cognitive deficits associated with this condition.

A key finding of the study is that positive affect moderates the relationship between pain severity and moderate physical activity but does not moderate the relationship between moderate physical activity and cognitive performance. This result suggests that positive affect plays a critical role in how pain influences participation in physical activities. Specifically, high levels of positive affect appear to strengthen patients’ ability to remain physically active despite pain. This is consistent with Fredrickson’s [13] theory on the benefits of positive affect in broadening the repertoire of thoughts and actions, which may include participation in physical activities despite pain.

A moderated mediation analysis revealed that moderate physical activity mediates the relationship between pain severity and cognitive performance, and this mediating effect is modulated by the level of positive affect. Specifically, bootstrap results show that mediation is significant when positive affect is one standard deviation below the mean. This finding is consistent with the “limited workspace” theory [7], which suggests that chronic pain consumes cognitive resources, leaving less available for other tasks. In this study, moderate physical activity improved cognitive performance only when positive affect levels were low. This indicates that positive affect may mitigate the impact of pain on physical activity and hence on cognitive performance. This result could be explained by several mechanisms. One possible explanation is that physical activity itself may act as a distraction mechanism that reduces pain perception, allowing cognitive resources to be freed up for other tasks [18]. However, when positive affect levels are high, this additional benefit of physical activity may not be as pronounced because positive affect is already providing a significant buffer against the impact of pain on cognitive resources. Furthermore, it is possible that positive affect itself is directly related to cognitive function through neurobiological mechanisms, such as increased neuroplasticity and the release of neurotransmitters that enhance cognitive performance [13,44,45]. Thus, individuals with high levels of positive affect may experience less cognitive decline due to chronic pain regardless of their level of physical activity [20].

The relationship between positive affect and physical activity adherence is complex and bidirectional. Not only do positive emotional states facilitate motivation to engage in physical activity, but exercise itself may induce improvements in positive affect. This positive cycle is crucial for patients with fibromyalgia, as adherence to long-term exercise programs can be difficult due to pain and fatigue barriers [22]. Interventions that foster both emotional well-being and physical activity may therefore offer synergistic benefits.

These findings have significant clinical implications. First, they underline the importance of not only promoting moderate physical activity but also of intervening to enhance positive affect in patients with fibromyalgia. Interventions such as cognitive behavioral therapy, mindfulness, and other strategies aimed at improving emotional well-being could complement physical exercise programs, thereby enhancing their beneficial effects on cognition.

Our findings also have important implications for future research. First, they suggest the need for longitudinal studies to investigate how variations in physical activity and positive affect may interact over time to influence cognitive performance in patients with fibromyalgia. Furthermore, it would be useful to explore whether specific interventions designed to increase positive affect can amplify the benefits of physical activity in this population [46].

Despite the relevance of the results found, the present study presents some important limitations to be taken into account. First, the sample was composed only of women, which may limit the generalization of the findings in regard to men with fibromyalgia. Second, the use of self-reported measures to assess positive affect and physical activity could introduce recall and social desirability biases. Future studies could benefit from the use of objective measures of physical activity and more robust assessments of positive affect. Third, the use of a single scale for each measure (pain, affect, physical activity, or cognition) may have limited the ability to fully capture the complexity and variability of these constructs. Fourth, although the Stroop test is an effective tool for measuring attentional capacity and cognitive inhibition, its results may not be fully translatable to other cognitive performance tasks. It is acknowledged that these factors may have influenced the findings and should therefore be taken into account when interpreting the results. In future studies, we plan to explore the use of multiple scales for each measure and the inclusion of other cognitive tests to provide a more comprehensive and diverse assessment of cognitive performance. These limitations underscore the need for further research in this field to confirm and extend the findings. Finally, it would be interesting for future research to take into account other variables such as the smoking history of the participants. Smoking can have a significant impact on both physical and mental health, and its inclusion could have provided a more complete view of the factors influencing our findings.

## 5. Conclusions

In conclusion, our results indicate that moderate physical activity will mediate the relationship between pain and cognitive performance more effectively in low-positive-affect contexts. These findings underscore the importance of considering both emotional and behavioral factors when designing interventions to improve cognitive function in patients with fibromyalgia. Promoting positive affect along with physical activity may be an effective strategy to mitigate the effects of chronic pain on cognition, thereby improving the quality of life of these patients [15].

## Figures and Tables

**Table 1:** Descriptives of psychosocial and clinical characteristics (*n* = 244).

Psychosocial Characteristics	Mean (SD)	2.	3.	4.	5.	6.	7.	8.	9.	10.	11.	12.
1. Severity pain	7.44 (1.80)	−0.077	−0.252 *	−0.234 *	−0.052	−0.043	0.087	0.017	−0.159	−0.043	0.092	0.091
2. Positive Affect	28.02 (8.92)		0.252 *	−0.082	−0.132	−0.203	−0.047	−0.014	−0.033	−0.110	−0.115	−0.401 **
3. Moderate Physical activity	1.27 (2)			−0.278 **	−0.051	−0.052	0.156	−0.124	0.041	0.025	0.105	0.102
4. Cognitive performance	−0.97 (10.87)				−0.214 *	−0.075	−0.231 *	0.009	0.070	0.095	−0.033	−0.095
5. Muscle relaxants	2.44 (3.17)					0.139 *	0.206 **	0.195 **	−0.065	0.180	0.056	0.129
6. Antidepressants	4.63 (3.31)						0.273 **	0.275 **	0.079	−123	0.217 *	0.272 **
7. Analgesics	5.54 (2.53)							0.259 **	−0.066	0.152	0.117	0.204 *
8. Sleeping pills	4.30 (3.32)								−0.001	0.068	0.191 *	0.241 **
9. Height (cm)	172 (13.3)									0.009	0.081	0.029
10. Weight	72.12 (16.31)										0.165	0.230 *
11. Anxiety	11.77 (3.82)											0.530 **
12. Depression	9.41 (4.25)											

* *p* < 0.05 y ** *p* < 0.01; abbreviations: SD (standard deviation), FM = fibromyalgia.

**Table 2:** Moderate mediation analysis assuming positive affect as moderator.

VD: Moderate Physical Activity	*R* ^2^	*F*	*p*	Beta	*t*	*p*
Model summary	0.46	2.45	0.022			
VI: Pain Severity				−0.52	−2.71	0.010
W: Positive Affect				−0.16	−1.64	0.108
Positive Affect × Pain Severity				0.02	2.42	0.018
Antidepressants (covariate)				0.07	0.89	0.388
Analgesics (covariate)				−0.03	−0.40	0.689
Depression (covariate)				0.06	1.60	0.112
**VD: Cognitive Performance**	***R* ^2^**	***F***	***p***	**Beta**	***t***	***p***
Model summary	0.47	2.80	0.013			
VI: Pain Severity				−0.93	−1.14	0.268
M: Moderate Physical Activity				3.02	2.20	0.030
W: Positive Affect				−0.51	−1.40	0.172
Positive Affect × Moderate Physical Activity				−0.07	−0.36	0.718
Antidepressants (covariate)				−7.15	−1.17	0.242
Analgesics (covariate)				−0.20	−0.08	0.931
Depression (covariate)				−0.09	−0.18	0.855

**Table 3:** Indirect conditional effect at specific levels of the moderator (positive affect) when treating moderate physical activity as a mediator.

Positive Affect	Beta	SE	LL 95% CI	UL 95% CI
1SD below the mean	−0.22	0.085	−0.42	−0.04
Mean	−0.06	0.061	−0.19	0.41
1SD above the mean	0.10	0.10	−0.08	0.31

Notes: SE = standard error; LL 95% CI = lower level of the 95% confidence interval; UL 95% CI = upper level of the 95% confidence interval.

## Data Availability

The data presented in this study are available on request from the corresponding author. The data are not publicly available due to privacy restrictions.
